# Manipulating two-dimensional colloidal crystals using Laguerre–Gaussian beams

**DOI:** 10.1039/d6sm00456c

**Published:** 2026-09-11

**Authors:** Habib Moradi, Sjors Cornielje, Mathieu G. Baltussen, Berend van der Meer, Arran Curran, Roel P. A. Dullens

**Affiliations:** a Institute for Molecules and Materials, Radboud University Heyendaalseweg 135 6525 AJ Nijmegen The Netherlands roel.dullens@ru.nl

## Abstract

Optical tweezing has firmly established itself as a versatile tool in fields ranging from biology to physics. In colloidal systems, optical tweezers are used to *e.g.* measure small forces or to generate optical potential energy landscapes. More complex and dynamic optical tweezing can be readily achieved using holographic wavefront engineering, though this is typically applied to only a handful of particles. Here, we discuss how we use holographically generated Laguerre–Gaussian (LG) beams to manipulate hundreds to thousands of particles in a two-dimensional colloidal crystal to form well-defined loop-shaped grain boundaries. We characterise the axial intensity distribution of the LG beam and show the effect of applying LG beams at different distances from the focal point to the colloidal crystals. In addition, we demonstrate how LG beams with different vortex numbers can be used to generate grain boundary loops with different radii, and finally we briefly compare and contrast manipulating 2D colloidal crystals with a single defocussed LG beam *versus* using a focussed LG beam combined with a defocussed Gaussian beam.

## Abstract 2

We demonstrate how a single defocused Laguerre–Gaussian beam can manipulate hundreds to thousands of particles in dense colloidal monolayers, exemplified by the controlled formation of grain-boundary loops in two-dimensional colloidal crystals.

## Graphical abstract

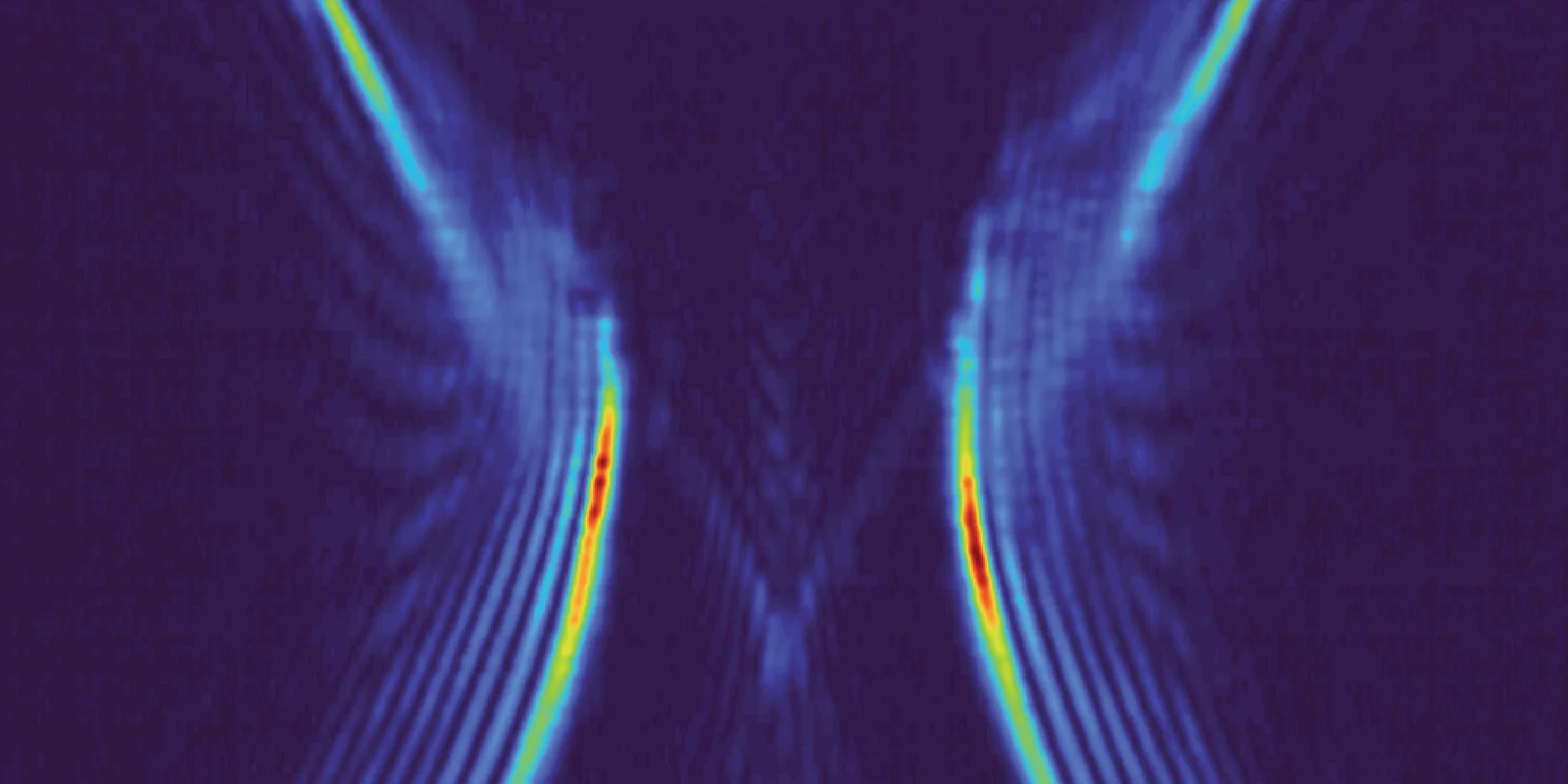

## 1 Introduction

Defects such as dislocations, vacancies, impurities, and grain boundaries critically influence the properties of a material, acting as structural discontinuities that affect stress relaxation, plasticity, and transport.^1–3^ Their local structure and mobility govern key phenomena such as recrystallisation, grain coarsening, and defect nucleation.^1–3^ In particular, grain boundaries act as barriers to dislocation motion and diffusion pathways, influencing mechanical strength, corrosion resistance, and overall material stability.^1–3^ In atomic systems, directly observing these defects remains challenging.^4,5^ Alternatively, colloidal systems offer a versatile platform where defects can be observed with relative ease.^6–9^ Uniform colloidal spheres, dispersed in a solvent will, under appropriate conditions, self-assemble into ordered crystalline structures^10^ which facilitates observing defects directly with optical microscopy techniques.^7–9^ As such, colloidal systems serve as powerful model systems for studying grain boundary structure and dynamics.^11–17^

An important additional advantage of colloidal systems is that they are readily manipulated using relatively small external forces.^18,19^ During the past few decades defects in colloidal crystals have been generated and controlled using various external forces. Electric fields have been used to induce and steer grain boundaries by tuning the dipolar interactions of charged colloids,^20^ while magnetic fields have been used to create and destroy defect structures in paramagnetic colloid systems.^21^ In microgravity environments or with density-matching solvents, gravity itself can be used to induce defect annealing through sedimentation,^22^ while crystallisation and grain growth under shear have also been studied.^17,23–25^ Confinement and tailored boundary conditions, for example using microfluidic devices,^26,27^ have likewise provided routes for controlling crystal structure and defect formation.

Optical tweezers offer an exceptionally versatile and non-invasive means of manipulating soft matter, using the optical forces of tightly focussed laser beams. Since their invention by Ashkin,^28^ optical traps have enabled precise, non-contact control over micron-sized particles in three dimensions, making them invaluable tools for probing interparticle forces, assembling colloidal structures, and studying defect dynamics.^29–31^ Conventional Gaussian optical tweezers excel at manipulating individual particles or small groups of particles.^8,32–35^ Irvine *et al.*^33^ first demonstrated the controlled creation of circular grain boundaries using an array of Gaussian optical traps to apply a torque to a small number of particles. Cash *et al.*^34^ subsequently employed optical tweezers to locally melt crystalline regions, resulting in the spontaneous formation of grain boundary loops (GBLs). Manipulating over extended regions containing hundreds or even thousands of interacting particles requires a different optical manipulation approach such as the use of speckle patterns.^36,37^

Structured light provides another natural route towards such collective manipulation. In particular, Laguerre–Gaussian (LG) beams possess a helical phase structure and carry orbital angular momentum,^38^ enabling optical torques to be transferred continuously over extended regions. When generated holographically using a spatial light modulator, LG beams offer a highly flexible means of tailoring both the spatial distribution of optical forces and the amount of angular momentum delivered to a sample.^39^ Beyond colloidal crystals, this capability has potential applications wherever controlled rotational forcing of dense soft matter is desirable, including studies of defect formation in crystalline soft materials, yielding and plastic deformation, optical fractionation, droplet manipulation, and other many-body systems.

For two-dimensional colloidal crystals, holographically generated LG beams provide a particularly elegant method for generating circular grain boundaries. Rather than manipulating particles individually, the optical torque is distributed over an extended annular region, causing an entire crystalline domain to undergo rigid-body rotation relative to the surrounding lattice. We previously demonstrated that a combination of a LG beam and a defocused Gaussian beam could rotate crystalline domains with radii of ∼10 particle diameters,^40,41^ producing GBLs with well-defined size and orientational mismatch. And more recently, we introduced the use of a single defocused LG beam to create significantly larger GBLs, with radii approaching ∼35 particle diameters, thereby enabling quantitative investigations of grain boundary migration^42^ and shrinkage^43^ in two-dimensional colloidal crystals.

Although the use of LG beams for manipulating colloidal crystals has been demonstrated, practical aspects are only briefly described in our earlier research articles.^40–43^ The aim of this article is to disseminate both the underlying principles and the practical implementation of holographically generated LG beams as a versatile experimental tool for manipulating dense soft matter. Using two-dimensional colloidal crystals as the primary example, we discuss the generation and characterisation of LG beams, the influence of beam parameters and focal position on the resulting optical force field, and the creation of GBLs with tuneable size through the use of different topological charges. More broadly, this article provides sufficient practical detail to enable the technique to be readily adopted and extended to a wide range of problems involving the controlled manipulation of condensed soft matter.

## 2 Introduction to Laguerre–Gaussian beams

**Fig. 1:**
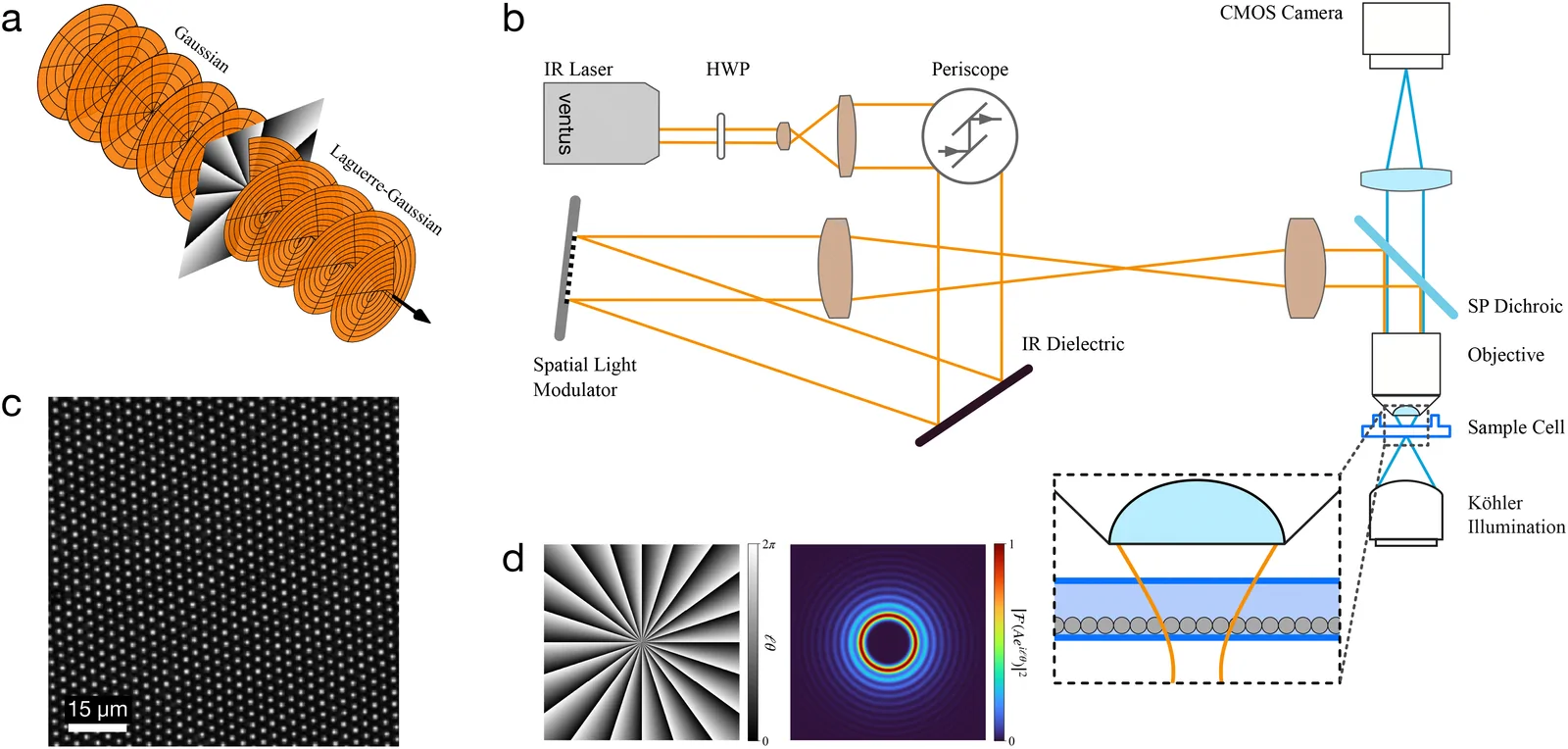
(a) A schematic showing how the hologram changes the wavefront of an incoming Gaussian beam into that of an Laguerre–Gaussian beam. (b) A schematic of our holographic optical tweezing setup combined with a brightfield microscope. The inset shows a zoom of the sample. (c) Microscopy snapshot of a two-dimensional colloidal crystal. (d) A spiral phase mask with a topological charge of *l* = 20 used to generate the Laguerre–Gaussian beam along with the predicted intensity profile.

Unlike the traditional Gaussian beam, which when collimated is approximately described by planar wavefronts, the LG beam is a unique optical mode with a helical wavefront (see Fig. 1(a)) that when focussed creates a ring-shaped intensity distribution with a central dark core. In this work we use the lowest radial order of the LG family, which consists of a single bright ring surrounding a dark core.^38^ The electric field in the sample plane can be written as^38^1
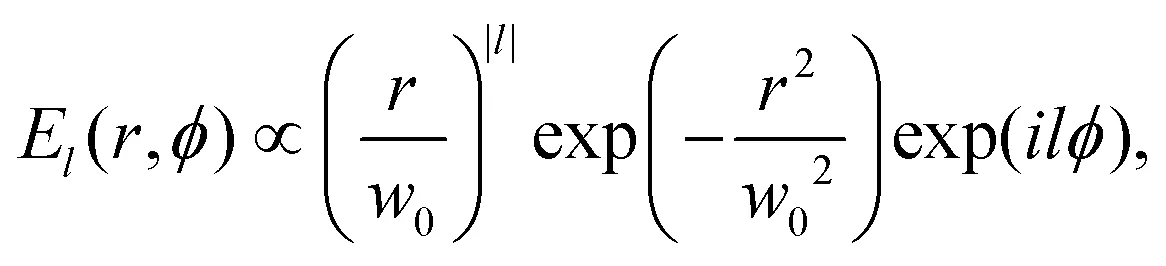
where *r* and *ϕ* are the radial and azimuthal coordinates, and *w*_0_ is the beam waist. The key parameter is the integer *l*, known as the vortex number or topological charge, which sets the number of 2π phase windings around the optical axis. The azimuthal phase term exp(*ilϕ*) creates this helical structure and leads to a central phase singularity, consistent with the general role of phase gradients in generating transverse optical forces and torques.^44,45^ The radial prefactor (*r*/*w*_0_)^|*l*|^ shifts the intensity maximum away from the centre, while the Gaussian envelope exp(−*r*^2^/*w*_0_^2^) sets the overall width. The characteristic radius of the ring increases with the vortex number, and under strong focusing conditions increases approximately linearly with |*l*|, *i.e. R* ∼ *l*.^39^ Since the photon flux is distributed along the circumference of the vortex, the intensity experienced by a particle at the ring scales approximately as *I*_0_ ∼ *P*/(2π*R*Δ*r*),^39^ where *P* is the laser power and Δ*r* is the radial width of the ring, which is independent of *l*.^46^ Each photon in an LG beam carries an angular momentum of *lħ*,^38,47^ which can be transferred to trapped particles.^48,49^ A simple scaling estimate suggests that the angular velocity *ω* of a trapped particle scales as 
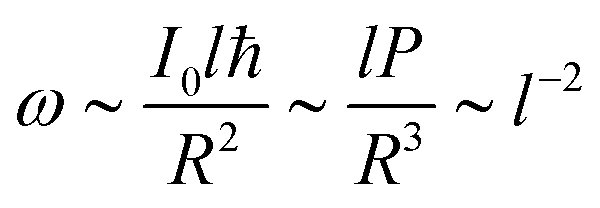
, illustrating how LG beams can be used to drive controlled rotational motion.

The vortex beams used in our experiments are generated with a phase-only spatial light modulator (SLM) using holographic optical trapping methods.^50,51^ The SLM is programmed with a spiral phase mask of the form^52^

2 *Φ*(*x*,*y*) = [*lϕ*(*x*,*y*) + *k*_*x*_*x*]mod 2π,

where (*x*,*y*) = (*i* − *i*_0_,*j* − *j*_0_) are the pixel coordinates relative to the centre pixel (*i*_0_,*j*_0_) of the SLM, and *ϕ*(*x*,*y*) = atan2(*y*,*x*) is the corresponding azimuthal coordinate with a topological charge *l*. The second term introduces a linear phase tilt that spatially separates the beam from the undiffracted (zeroth-order) beam.^53^ When this phase mask is illuminated by a collimated Gaussian beam, the resulting beam exhibits the characteristic helical wavefront of an LG beam, as shown in Fig. 1(a). By varying the topological charge *l* and the optical power, we control the orbital angular momentum transferred to the colloidal particles and the resulting optical torque, which sets the scale and strength of the rotational field used to manipulate the two-dimensional crystal.

## 3 Experimental methods

### 3.1 Optical system

The general implementation of holographic optical tweezer systems has been discussed extensively elsewhere.^54–56^ Here, we focus on the practical considerations specific to our implementation, which are dictated by the need to capture relatively large fields of view (FoV) (∼600 × 600 µm^2^) in order to capture large circular grain boundaries. The trade-off for modest magnification and large FoVs, is the low numerical aperture (<1). This limits an optical trap to 2D trapping due to the axial scattering force overpowering the gradient force, resulting in colloids being pushed in the direction of propagation, out of the trap. We note however that three-dimensional trapping with a single Laguerre–Gaussian beam has been reported.^57^

Fig. 1(b) shows the complete experimental setup, including the optical path used to generate and relay the holographic LG beam and the microscope used for particle manipulation and imaging. In our 2D optical trapping system we use an upright microscope configuration so that we are trapping from above and the bottom wall of the sample cell helps to form a stable optical trap (see Fig. 1 (b, inset)). In principle, other geometries could also be used, *e.g.* an inverted geometry, though the presence of a wall or fluid–fluid interface^58^ is important to minimise the effect of the axial scattering force, which allows for efficient in-plane manipulation of the system by the LG beam. The trapping system is based on an infra-red laser with a wavelength of 1064 nm, which is expanded and collimated before being reflected off a phase-only reflective SLM (1920 × 1152 pixels, 9.2 µm pixel pitch, Meadowlark Optics HSP1920-1064). The phase modulated beam is then relayed through a 4f optical system consisting of two achromatic lenses with focal lengths of 400 mm and 250 mm for the first and second lenses after the SLM, respectively, in order to form an image of the hologram at the back focal plane of the microscope objective. The choice for the focal lengths of these two lenses is determined by the hologram size and microscope objective's back aperture diameter, such that the hologram image overfills the aperture.^54^

The imaging system employs a bright-field microscope using Köhler illumination to ensure uniform sample illumination. The sample is imaged using a 40× microscope objective with a numerical aperture of 0.4, paired with a 180 mm focal length tube lens. Images are captured using a CMOS camera (2048 × 2048 pixels) with a maximum frame rate of 90 Hz. The imaging optics were selected to balance magnification and FoV, ensuring suitable images for particle tracking algorithms whilst achieving a FoV large enough to capture large circular grains with a maximum radius of ∼300 µm.

### 3.2 Colloidal system

In principle, our method can be applied to any (soft matter) system suitable for manipulation by optical trapping. The sample composition and preparation required for optical trapping depend on the soft-matter system under investigation and the intended optical manipulation. General considerations for sample preparation and optical trapping are discussed in detail elsewhere.^54,59^ Our colloidal system consists of *σ* = 2.84 µm diameter melamine spheres (microParticles GmbH), dispersed in Milli-Q water and ethanol (80 : 20 v/v), the latter serving to suppress bacterial growth. The sample is pipetted into a quartz cell (20 mm × 9 mm × 200 µm, Hellma) and sealed to prevent solvent evaporation. At high area fractions (>0.70), the particles form colloidal crystallites,^60^ which eventually coarsen into larger single crystals over a period of 24 hours, see Fig. 1(c).^16^ Note that the particles' polydispersity should be below ∼10% for 2D hard spheres crystals to form^61^ and that the freezing transition also depends on the interaction potential.^62–64^ While our LG beam method is applied to a gravitationally confined 2D colloidal system, where the gravitational length (0.068 µm in our case) has to be much smaller than the particle diameter,^60^ it is in principle also compatible with other sample geometries where the colloidal system is confined to 2D by, for instance, two glass slides^65^ or a fluid–fluid interface.^58^

### 3.3 Data acquisition and image analysis

The position of all individual colloidal particles is obtained from the bright-field images using a standard centroid-tracking routine,^66^ in this case Trackpy.^67^ This gives the (*x*,*y*) position of every particle in each frame. From these coordinates we extract the local crystalline orientation and, in particular, the relative misorientation between neighbouring regions in the monolayer. The analysis code, together with example data sets, is publicly available in the accompanying GitHub repository.^68^

To quantify the local crystalline order we calculate the complex hexagonal order parameter *ψ*_6_(**r**_*j*_) for each particle. The nearest neighbours are identified from the Delaunay triangulation of the particle coordinates. For a particle *j* with *N*_*j*_ neighbours we define^69–71^3
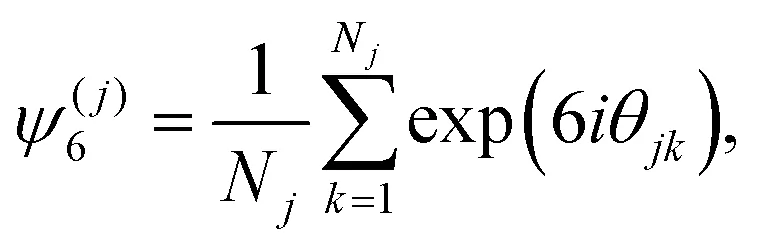
where *θ*_*jk*_ is the angle between the bond between particles *j* and *k* and the laboratory *x*-axis. In a well-ordered hexagonal region |*ψ*^(*j*)^_6_| approaches unity, while near defects or distorted regions its value decreases. The phase of *ψ*^(*j*)^_6_ contains the local bond-orientational information. We determine the orientation of a particle *j*, *θ*^(*j*)^_6_, *via*^72^4
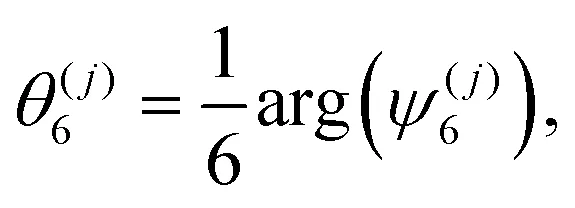
where the orientation falls within interval [0,60°), consistent with the sixfold symmetry of the hexagonal lattice. Differences in *θ*_6_ between neighbouring crystalline regions, *i.e.* across grain boundaries, thus represent the local misorientation. For a GBL, this corresponds to the difference in orientation between the rotated inner crystalline patch and the surrounding crystal. To reduce noise caused by thermal fluctuations, we smooth the *θ*_6_ field by averaging *ψ*^(*j*)^_6_ over the particles belonging to the first and second neighbour shells of particle *j*, following the approach used for polycrystalline monolayers.^16^ Using the smoothed orientation field, grain boundaries and their misorientations are readily found.^16^

Finally, we characterise the local packing fraction using a Voronoi construction, where each particle is assigned a polygonal Voronoi cell with an area *A*_*j*_. The local area fraction is then defined as5
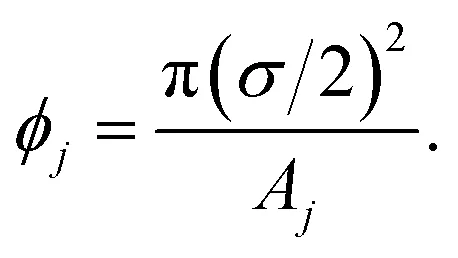


Note that for consistency with the bond-orientational analysis, we also smooth the local area fractions over the first two coordination shells.

## 4 Results and discussion

### 4.1 LG beam profile and intensity distribution

**Fig. 2:**
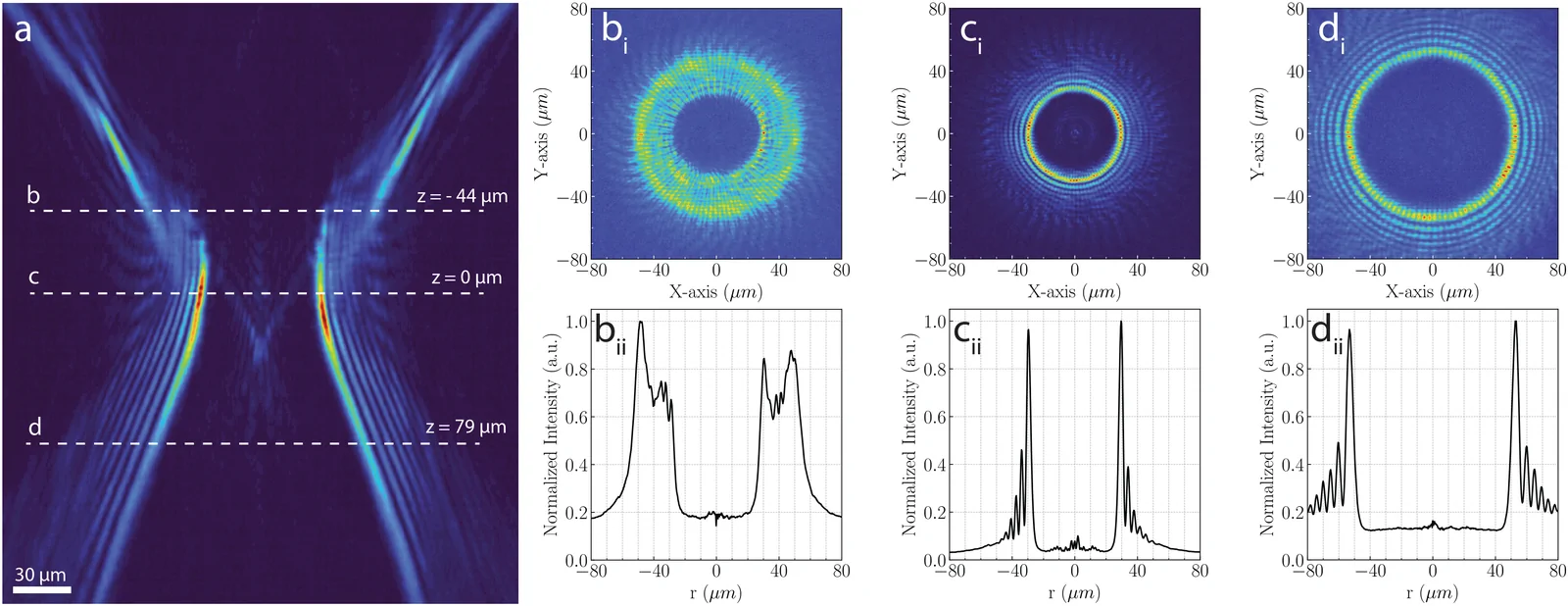
The intensity distribution of the Laguerre–Gaussian beam. (a) Complete beam profile showing the Laguerre–Gaussian beam intensity as it propagates from top to bottom through the focal region. (b_i_), (c_i_) and (d_i_) Intensity distributions in the planes before, at, and after the focal point, respectively, with corresponding radial intensity profiles shown below each panel.

In this section, we characterise the intensity distribution of the LG beam generated by our optical system. To obtain the beam profile at the sample plane, we place a mirror in the focal plane of the objective and image the resulting in-plane intensity distribution using the camera. Using a piezo stage we then move the mirror in the *z*-direction, *i.e.* along the optical axis, thereby building up a 3D image of the beam intensity.^73^Fig. 2(a) shows an orthogonal (*x*–*z*) view of the normalised intensity of an LG beam with vortex number of *l* = 50, obtained by scanning the mirror in 2 µm steps. As the beam propagates from top to bottom, it is converging towards its focus after which it diverges again. The dashed lines *b*, *c* and *d* indicate axial positions of a plane before, at and after the focal plane, respectively, and the corresponding intensity distributions are shown in Fig. 2b_i_, c_i_ and d_i_. The intensity cross-sections (rotationally averaged over an angle of π) are also shown underneath each panel. Clearly, before the focal plane, the LG beam exhibits a broad donut-like intensity distribution, whilst at the focal plane the LG beam exhibits a sharp ring-like intensity distribution, the radius of which increases beyond the focal plane.

### 4.2 Interaction of LG beam with dense colloidal systems

Next, we examine how the LG beam interacts with a dense 2D colloidal system. In particular, we explore the effect of the LG beam on the particle motion and deformation of a crystalline monolayer of colloidal particles and show how the colloidal particles respond to the LG beam upon varying different parameters such as the distance from the focal plane of the LG beam, the laser power, and the vortex number of the LG beam.

#### 4.2.1 LG beam before, at, and beyond the focal point

**Fig. 3:**
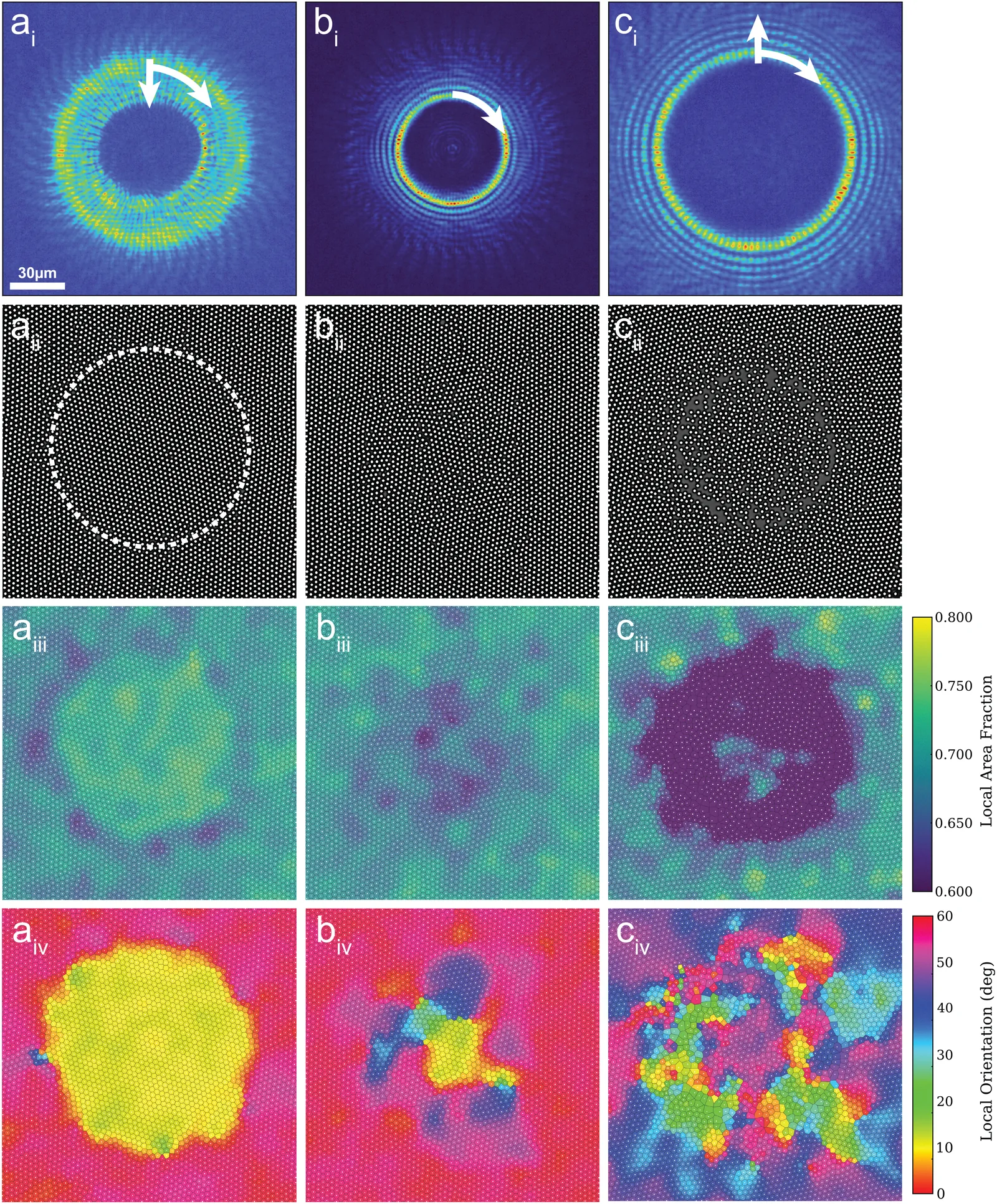
(a_i_), (b_i_) and (c_i_) Intensity distributions and force components of a Laguerre–Gaussian beam with *l* = 50 at different positions relative to the focal point: (a_i_) before, (b_i_) at, and (c_i_) after the focal point. White arrows indicate the direction of forces acting on particles. (a_ii_), (b_ii_) and (c_ii_) Corresponding microscopy snapshots of the colloidal monolayer after exposure to the Laguerre–Gaussian beam. (a_iii_)–(c_iii_) Local area fraction (*ϕ*_*j*_) maps and (a_iv_)–(c_iv_) local orientation (*θ*^(*j*)^_6_) angle maps of the colloidal crystal (see Section 3.3) after the interaction with the LG beam.

First, we discuss how the LG beam at three different axial positions (shown in Fig. 2) interacts with the colloidal crystal. To this end, we again show the intensity distribution at these planes in Fig. 3(a_i_), (b_i_) and (c_i_), but now labelled with white arrows to indicate the direction of the optical forces applied to the particles. The resulting deformations of the colloidal crystal at these axial positions are shown in the corresponding microscopy images underneath the intensity distributions. As shown in Fig. 3(a_ii_) only the LG beam at an axial position before the focal plane results in a block rotation of the crystal patch inside the optical vortex. At the focal plane (Fig. 3(b_ii_)) only the particles located at the ring of the highest intensity are being rotated, which results in partial melting of the crystal. If the focus is moved beyond the focal plane (Fig. 3(c_ii_)), the particles are being pushed radially outwards from the optical vortex causing a depletion of particles around the LG ring and melting of the crystal inside it.

These observations are consistent with the fact that the photons in an LG beam carry orbital angular momentum, which results in a rotational force being applied to the particles. However, in the case of defocussed LG beams the radial force component, which acts perpendicular to the beam axis in the radial direction, also comes into play (see radial arrows in Fig. 3(a_i_) and (c_i_)). Before the focal plane (Fig. 3a_i_ and a_ii_), the beam is converging and particles also experience an inward radial force that pushes them towards the optical axis. The resulting compression of the crystalline patch located inside the optical vortex is crucial to achieve a block rotation of the crystal that results in a well-defined GBL, as it prevents the break-up of the crystal during rotation. At the focal point (Fig. 3b_i_ and b_ii_), the beam is tightly focussed and the inward radial force component is negligible, with mainly the azimuthal (orbital) force dominating.^44,45^ Beyond the focal point, the beam starts diverging and particles experience an outward radial force that pushes them away from the beam axis (Fig. 3c_i_ and c_ii_). These observations indicate that it is the combination of the azimuthal (orbital) and radial forces that determines how an LG beam interacts with our 2D colloidal crystal, and that this interaction depends sensitively on the relative distance to the focal point.

This interpretation is further supported by quantifying the structural changes in the colloidal crystal using the analysis methods described in Section 3.3. The local area fraction (*ϕ*_*j*_) maps shown in Fig. 3(a_iii_), (b_iii_) and (c_iii_) reveal how the packing density varies across the crystal after manipulation by the LG beam. Clearly, before the focal plane (Fig. 3(a_iii_)), the area fraction of the rotated region is slightly higher than the outer crystal, demonstrating the compression due to the radially inward optical forces. At the focal plane (Fig. 3(b_iii_)), the area fraction is more-or-less constant, while beyond the focal plane (Fig. 3(c_iii_)), the area fraction is significantly lower than the outer crystal, consistent with the radially outward optical forces. The corresponding local orientation (*θ*^(*j*)^_6_) maps are shown in Fig. 3(a_iv_), (b_iv_) and (c_iv_). Clearly, only for the LG beam before the focal plane (Fig. 3(a_iv_)), is a local block rotation achieved, as is evident from the constant local orientation of the inner patch, whilst at (Fig. 3(b_iv_)) and beyond (Fig. 3(c_iv_)) the focal plane the crystal locally melts, resulting in small crystalline patches. Together, our results show that only converging LG beams, *i.e.* at axial positions before the focal point, provide the combination of azimuthal rotation and inward radial pressure required to obtain well-defined and reproducible GBLs.

#### 4.2.2 Dependence of angular velocity on distance from the focal point

**Fig. 4:**
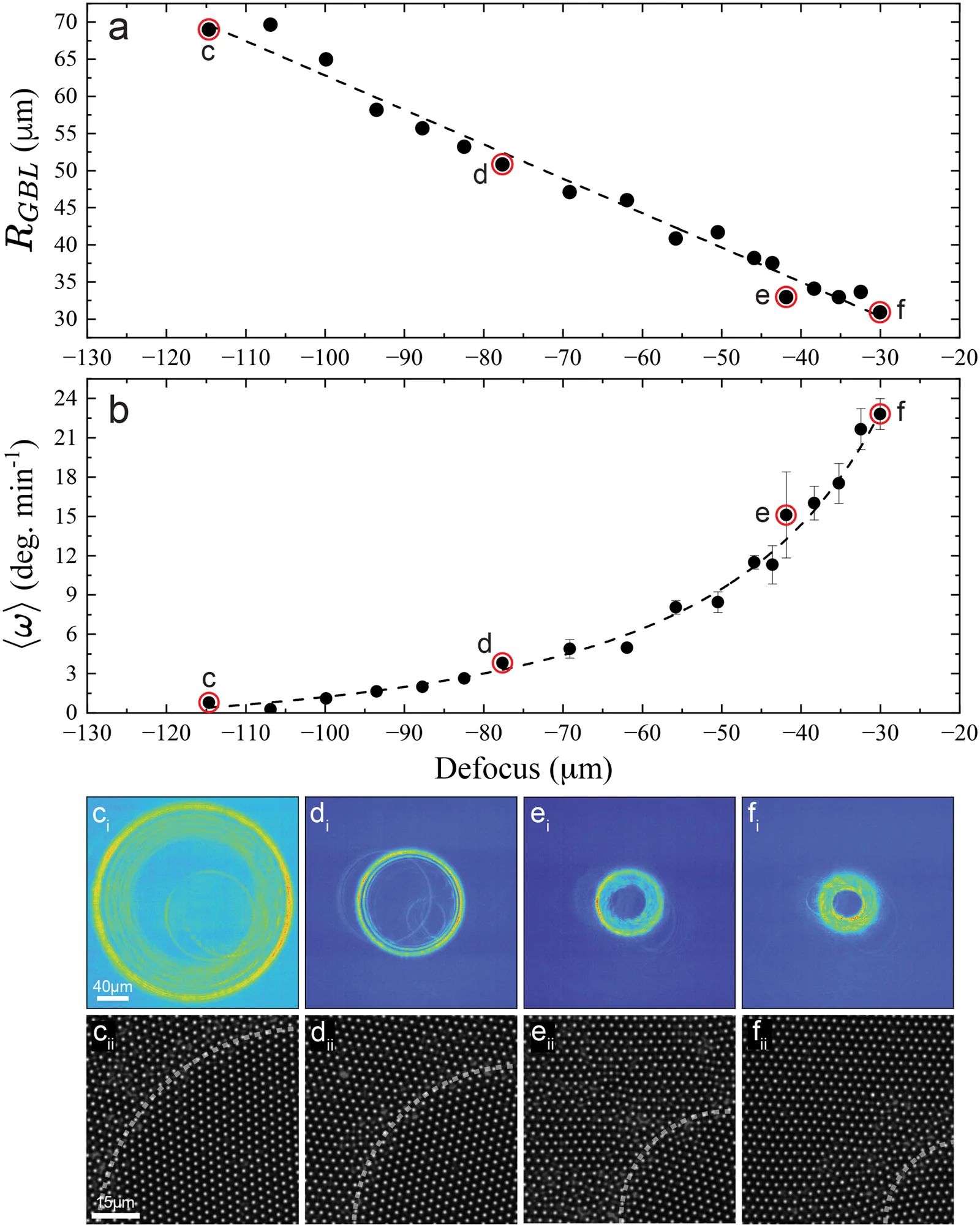
(a) Radius of the grain boundary loop (*R*_GBL_) as a function of distance from the focal point. (b) Average angular velocity of particles as a function of distance from the focal point. Vortex *l* = 30, laser power = 46.5 mW. (c_i_)–(f_i_) Normalised laser intensity distributions at four distances from the focal point. The bottom panel shows snapshots of the final state of the 2D crystal corresponding to these intensity distributions. For the angular velocity data, each point represents the mean over *N* tracked particles within the GBL from one movie. Error bars show the standard deviation across the particle values within that experiment.

Next, we characterise the angular velocity induced by LG beams at different axial positions before the focal point, *i.e.* within the converging-beam regime where the inward optical force results in rigid-body rotation of the inner crystal. In Fig. 4a we first show the radius of the resulting GBL (*R*_GBL_) as a function of the distance from the focal point while keeping the laser power constant, which is seen to be linearly dependent upon the defocus distance *d*, where *R*_GBL_ decreases upon approaching the focal point (at 0 µm). Fig. 4b shows the average angular velocity of the particles as a function of the same defocus distance. Starting from positions before the focal point, *i.e.* with a converging LG beam, the angular velocity of the particles increases as the beam approaches the focal point. Note that the reported angular velocity is the mean of these time-averaged values over all *N* particles included in the analysis, 〈*ω*〉. The error bars in the figures show the standard deviation across the particle values within the same experiment. Each data point was obtained from one movie and therefore the error bars represent particle-to-particle variation, rather than variation between repeated experiments.

The increase in angular velocity shown in Fig. 4b can be rationalised by the fact that far before the focal point, the laser power is distributed over a relatively large vortex-ring circumference, which results in a relatively low angular velocity. Upon moving closer to the focal point, the same laser power is distributed around a smaller vortex-ring circumference, which results in an increasing angular velocity. This is shown by the snapshots of the normalised laser intensities at the four different distances from the focal point in Fig. 4(c_i_)–(f_i_). More quantitatively, using that the radius of the grain boundary loop – as set by the radius of the LG beam *R* – is proportional to the defocus distance *d* and that *ω* ∼ *l*^−2^ and *R* ∼ *l* (see Section 2), the angular velocity is expected to scale as *ω* ∼ *d*^−2^, as indeed observed (see dashed line in Fig. 4b).

We note that for defocus distances up to ∼−30 µm the particles inside the optical vortex still form a crystal that exhibits rigid-body rotation, as shown by the snapshots of the final state (*i.e.* after rotation) of the 2D colloidal crystal in the bottom panel of Fig. 4. However, upon moving sufficiently close to the focal point, *i.e.* for our system beyond −30 µm, the rigid-body rotation of the inner crystal is eventually lost due to insufficient inward optical force.

#### 4.2.3 Dependence of angular velocity on laser power

**Fig. 5:**
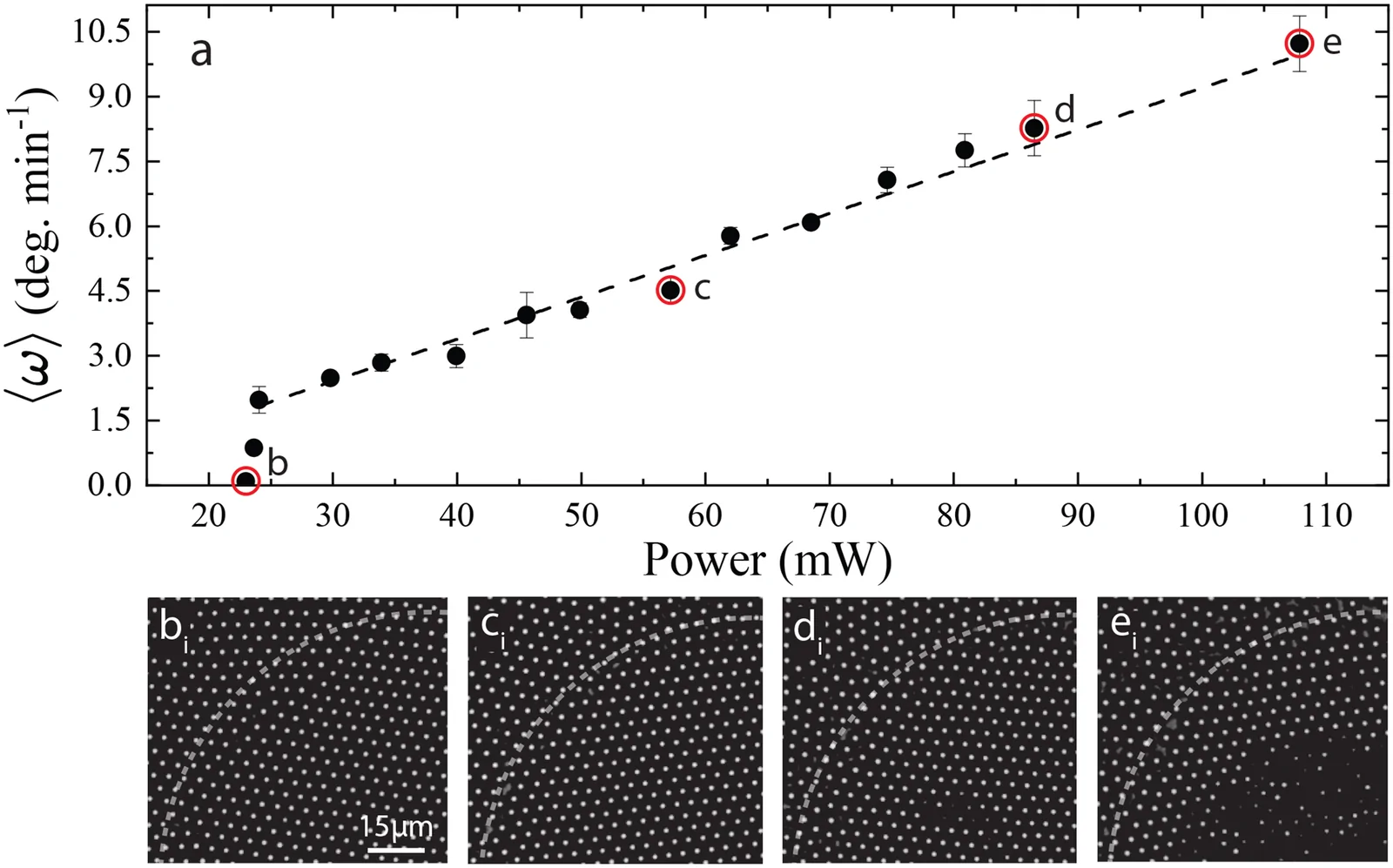
(a) Average angular velocity as a function of Laguerre–Gaussian beam power with a vortex number *l* = 30. The bottom panel shows snapshots of the final state of the 2D crystal for the laser power values in panels (b)–(e), as indicated in panel (a). For the angular velocity data, each point represents the mean over *N* tracked particles within the GBL from one movie. Error bars show the standard deviation across the particle values within that experiment.

Another factor that we can tune is the laser power, which is directly related to the intensity of the beam and the force it can apply to the particles. Fig. 5(a) shows how the average angular velocity of the particles depends on the power of the LG beam, with the vortex charge *l* = 30 and distance from the focal point held constant (*z* = −78 µm). Fig. 5(b_i_)–(e_i_) show the corresponding snapshots of the final state of the colloidal crystal system after manipulation by the LG beam with different laser powers as indicated in panel (a). Clearly, the angular velocity of the system shows a linear dependence on the incident beam power, as expected from the corresponding linear increase in photon flux and hence optical forcing. However, before this linear regime there is a minimum laser power required to achieve rotation – here around 23 mW – which we associate with the onset of yielding of the colloidal crystal. At higher laser powers, we also increase the inward optical force on the particles, which further compresses the particles and eventually pushes some of them out of the plane, resulting in the formation of a second layer of particles (see Fig. 5d_i_ and e_i_). In practice, this out-of-plane motion sets the upper limit on the laser power that can be used to generate well-defined GBLs in our 2D colloidal crystal.

#### 4.2.4 Dependence of angular velocity on vortex number

**Fig. 6:**
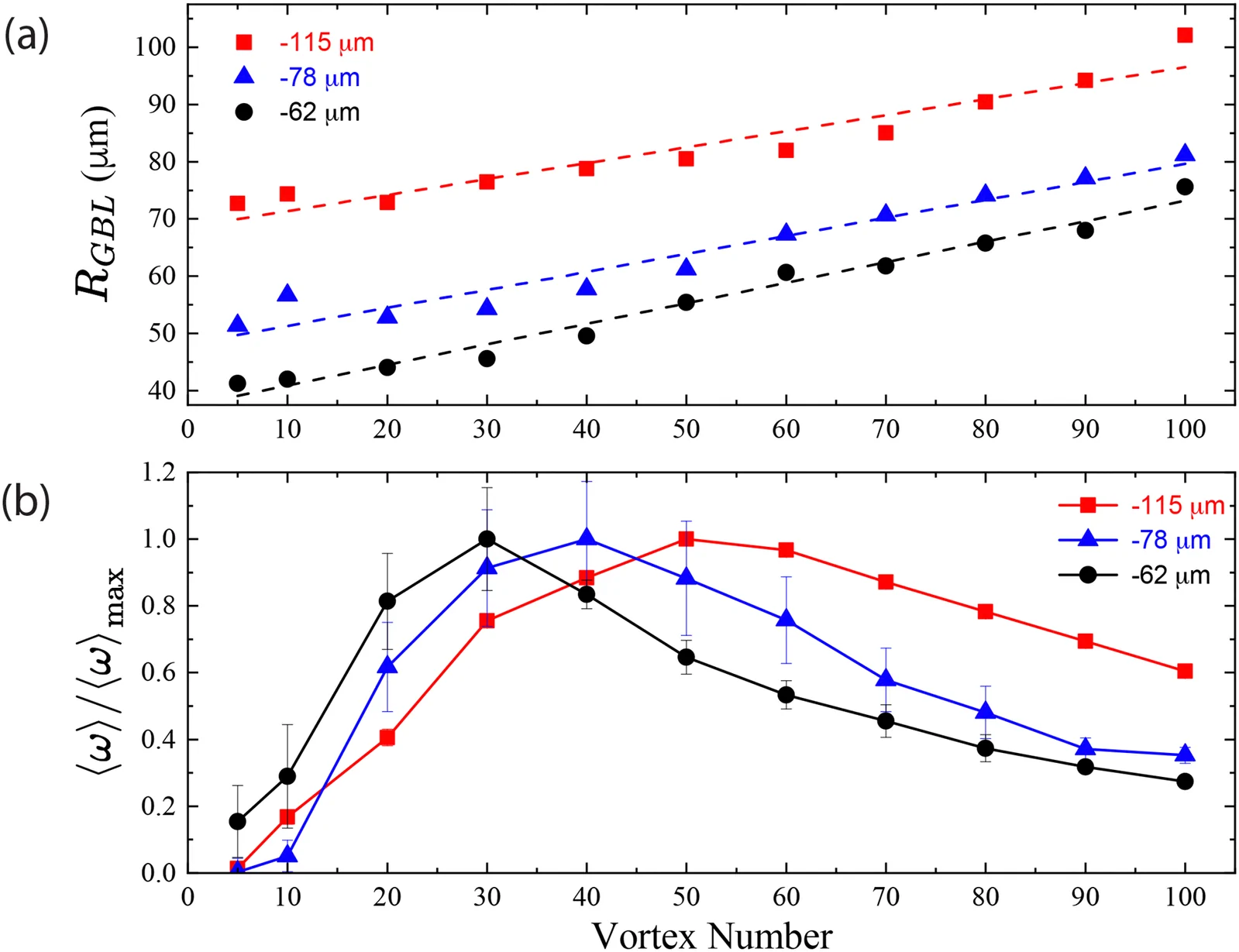
(a) Radius of the grain boundary loop (*R*_GBL_) as a function of vortex number for three different defocusing distances (still in the converging regime). (b) Normalised average angular velocity of the particles (each curve divided by its maximum value) as a function of vortex number of the beam, for the same defocusing distances. Together, the curves show how the position of the maximum angular velocity and the corresponding grain boundary loop size shift with defocus, reflecting the interplay between vortex size and available power density at the ring. For the angular velocity data, each point represents the mean over *N* tracked particles within the GBL from one movie. Error bars show the standard deviation across the particle values within that experiment.

The size of the LG beam can be conveniently tuned *via* its vortex number *l*. Fig. 6(a) shows the corresponding radius of the resulting GBLs as a function of vortex number for three different defocusing distances. The GBL radius increases approximately linearly with the vortex number, *i.e. R* ∼ *l*, as expected.^39^ To characterise how this affects the rotation of crystalline patches in the 2D crystal, we examined the dependence of the angular velocity on the vortex number *l*. Fig. 6(b) shows the normalised average angular velocity (*i.e.* divided by the maximum value for each curve) as a function of the vortex number for three different defocusing distances (note that these are all converging LG beams). The data reveal a clear non-monotonic behaviour, where at each defocusing distance there is a maximum angular velocity at a different vortex number. As the distance from the focal point increases, the vortex number corresponding to the maximum angular velocity shifts to higher *l*-values, indicating that the relationship between vortex number and rotation efficiency depends strongly on the beam's focal position.

The decrease of *ω* at large *l* can be rationalised by the scaling *ω* ∝ *l*^−2^ (see Section 2), which explains the reduction in angular velocity at large *l*: as the vortex number increases, the same optical power is distributed over a larger vortex-ring circumference, reducing the torque per particle. Additionally, corrugations in the intensity distribution – arising from the pixellated nature of the SLM – become more pronounced with increasing *l*, introducing approximately 2*l* azimuthal intensity modulations around the ring,^39^ which introduces additional resistance to particle motion and slows down the rotation. For small *l*, *i.e.* where the angular velocity increases with *l* (Fig. 6(b)), we observe a different mechanism.^39^ For small vortex numbers, the vortex ring is too small to provide a well-defined intensity distribution. The curvature of the helical phase front becomes too sharp, and the intensity profile becomes less uniform. These factors reduce the efficiency of torque transfer and hinder the formation of a rigidly rotating patch, even though the photon flux per particle is relatively high. In other words, the vortex is too small to efficiently trap and rotate a rigid crystalline patch.

The vortex number corresponding to the maximum angular velocity corresponds to the balance between these two competing effects: the vortex must be large enough to provide a well-defined intensity distribution for efficient torque transfer, but small enough to maintain sufficient power density for effective particle manipulation. This “optimal” value clearly depends on the distance to the focal point, as shown in Fig. 6. As the beam is more defocussed, the “optimal” vortex number shifts to higher values, reflecting the need for larger vortex rings to maintain effective particle manipulation at greater distances from the focal plane.

### 4.3 Combined Gaussian–LG beam method for circular grain boundary formation

In our earlier work,^40,41^ we introduced the use of LG beams to generate GBLs in 2D colloidal crystals. In those studies, however, we produced relatively small loops, with radii on the order of ∼10 particle diameters, using a combination of a focussed LG beam and a defocussed Gaussian beam. More recently, much larger GBLs, with radii of up to ∼35 particles, were achieved using defocussed (and converging) LG beams, as described in the present work.^42,43^ In this section, we briefly compare these two approaches and discuss their practical differences.

**Fig. 7:**
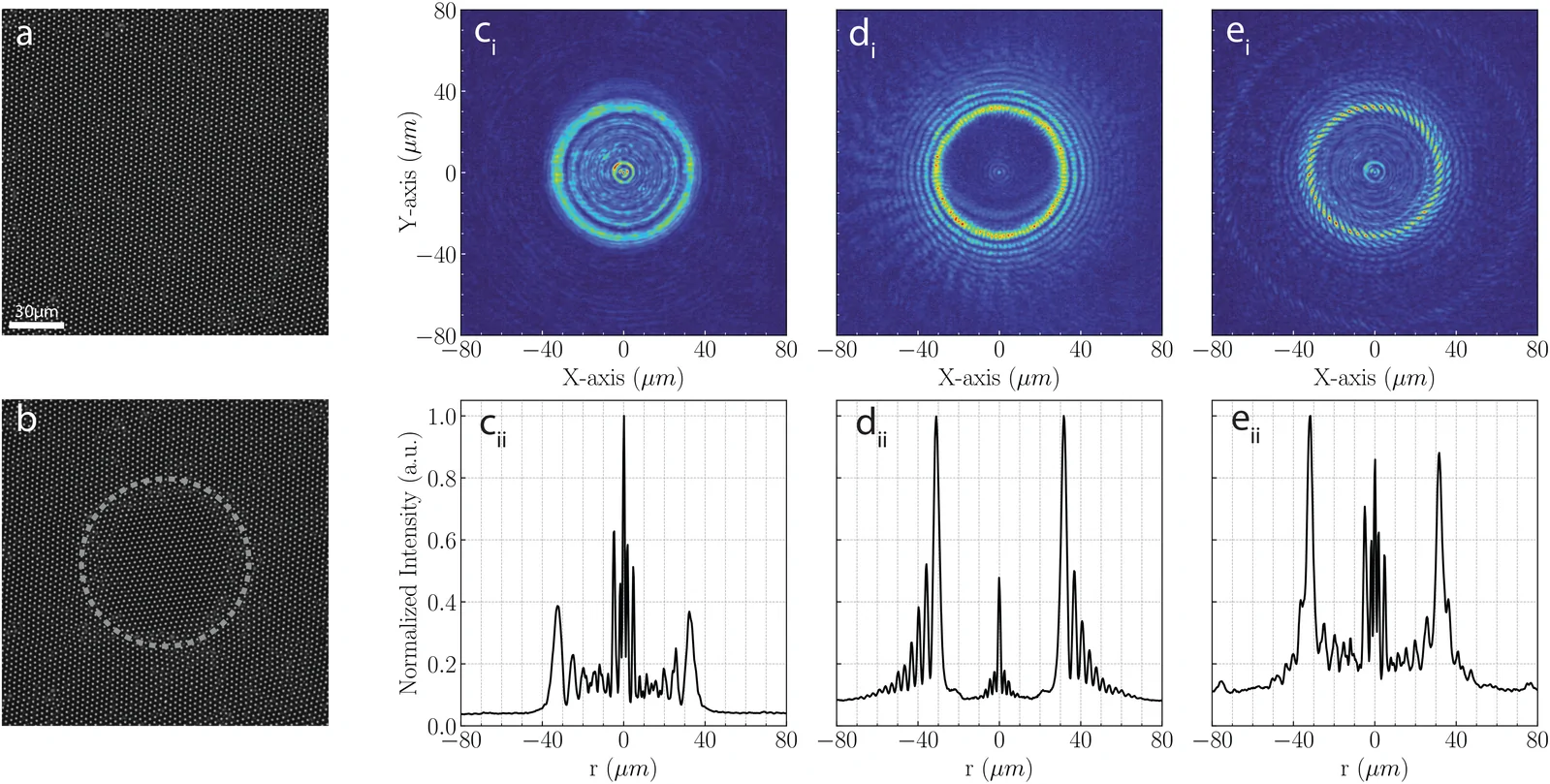
Manipulating the 2D colloidal crystal using a combination of a defocussed Gaussian beam and a focussed Laguerre–Gaussian beam. (a) and (b) Microscopy images of a two-dimensional colloidal crystal, with panel (b) showing a dashed circle indicating the region of optical manipulation. (c_i_), (d_i_) and (e_i_) Two-dimensional lateral intensity distributions of a defocussed Gaussian beam, a focussed LG beam with vortex number *l* = 50, and their combination, respectively. (c_ii_), (d_ii_) and (e_ii_) Corresponding one-dimensional radial intensity profiles for each beam configuration.

The combined LG–Gaussian beam method^40,41^ is relatively easy to implement and is capable of inducing rigid-body rotation in 2D colloidal crystals,^41^ as also demonstrated in Fig. 7a and b. However, it presents several limitations that make it less controllable than the present approach based solely on defocussed LG beams. A key difference lies in how the optical forces are generated. In the combined method, the roles of confinement and rotation are separated: the defocussed Gaussian beam provides an inward, compressive force that holds the particles together, while the focussed LG beam provides the azimuthal driving force responsible for rotation. This can be seen clearly in Fig. 7c_i_ and d_i_, where the Gaussian beam shows a central intensity peak, while the LG beam exhibits a ring-shaped intensity profile with a dark central core. Their combination in Fig. 7e_i_ produces a modified intensity distribution that merges these two effects, as also shown by the radial profiles in Fig. 7c_ii_, d_ii_, and e_ii_.

For this approach to work reliably, the radial sizes of the two beams must be closely matched. From the intensity distributions in Fig. 7c_i_ and d_i_, and the corresponding radial profiles in Fig. 7c_ii_ and d_ii_, it is evident that the characteristic radius of the Gaussian beam should correspond to the radius of the bright ring of the LG beam. If these sizes differ significantly, the particles experience spatially varying forces as they rotate, leading to fluctuations in the local confinement and torque. This can introduce irregular rotation, internal shear, and in some cases non-rigid-body motion or partial disruption of the crystal.

A second critical parameter is the intensity ratio between the two beams. Fig. 7e_i_ shows the combined two-dimensional intensity distribution, while Fig. 7e_ii_ shows the corresponding radial profile. If the intensity ratio is altered excessively, the combined radial profile in Fig. 7e_ii_ begins to resemble one of the two components: either the Gaussian profile, dominated by inward pressure, or the LG profile, dominated by rotation. In such situations, one effect becomes too strong relative to the other, resulting in either insufficient rotational synchronisation of the particles or weak confinement, both of which degrade the quality and stability of the rotating crystal domain.

Both of these constraints – matching the radial sizes and tuning the relative intensities – are absent in the defocussed LG beam approach used in the present work. Since both confinement and rotation originate from a single beam, there is no need for size matching or fine-tuning of relative intensities, leading to a more robust and controllable formation of large circular GBLs.

### 4.4 LG-beam mediated crystallisation

**Fig. 8:**
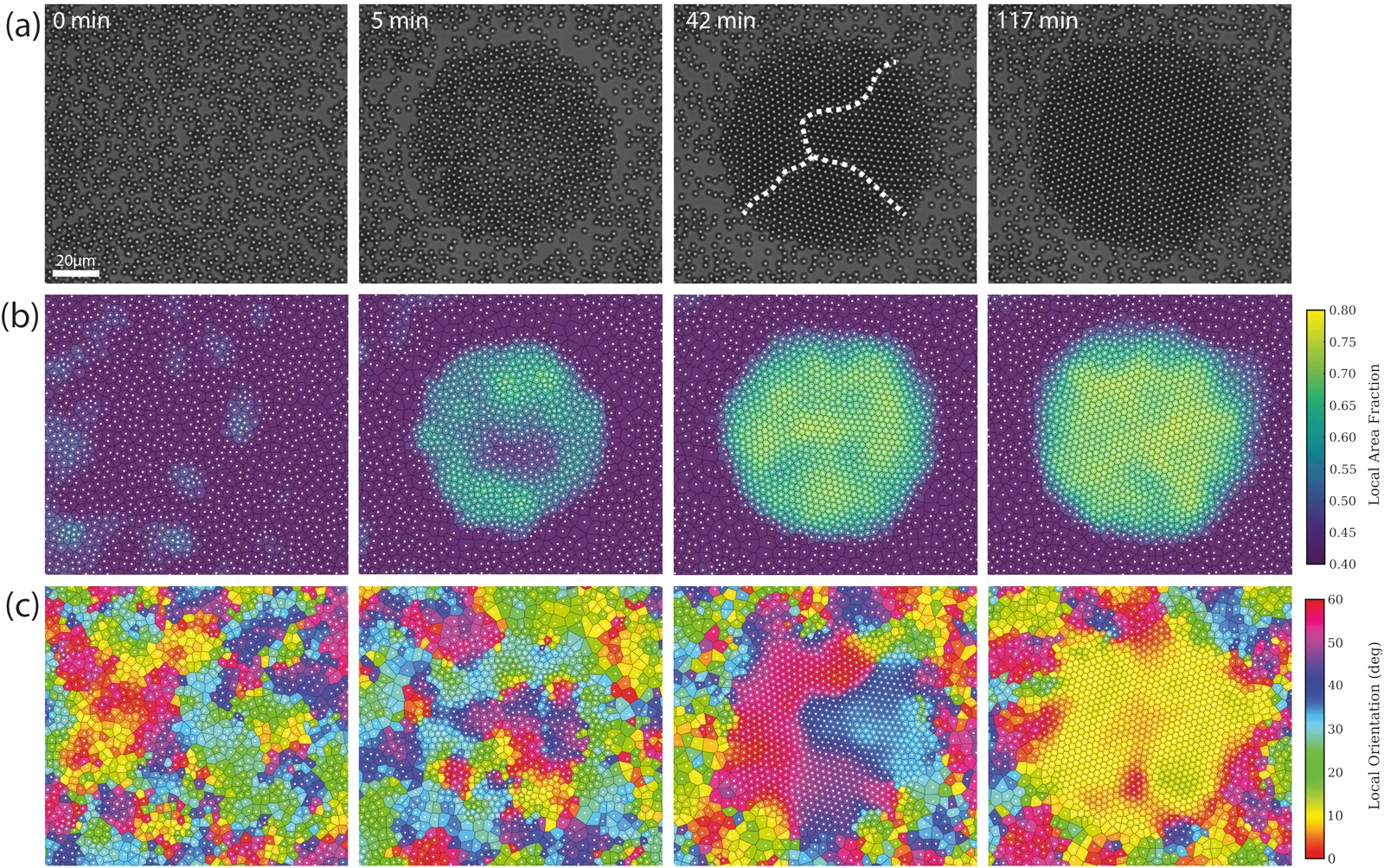
Time evolution of crystal nucleation and growth in a dilute colloidal system under Laguerre–Gaussian beam illumination. (a) Microscopy images showing the particle positions at different time points: 0 min, 5 min, 42 min, and 117 min. The dashed line at 42 min shows the grain boundaries present in the crystal. (b) Local area fraction (*ϕ*_*j*_) maps calculated from Voronoi tessellation at the same time points. (c) Local orientation (*θ*^(*j*)^_6_) maps showing the orientational order at the same time points.

Finally, we briefly demonstrate another application of our defocussed LG beam method by using it in dilute colloidal systems to initiate and control crystallisation. Our approach simultaneously provides optical confinement and rotational driving. Fig. 8 shows the time evolution of crystal nucleation and growth in a dilute colloidal system under the influence of a defocussed LG beam. The initial state consists of randomly distributed particles in a liquid-like configuration, as shown in Fig. 8 (a, 0 min). Once the LG beam is applied, particles are drawn into the circular region by the inward component of the optical force, while simultaneously experiencing orbital-angular-momentum-driven rotation. As a result, the area fraction inside the LG beam increases, leading to crystallisation, while the continuous rotational driving affects the crystallisation kinetics through ongoing particle rearrangements. As shown in Fig. 8 (a, 5 min and 42 min), multiple small crystalline domains with different orientations form, characteristic of the early stages of crystal nucleation. The local area fraction (*ϕ*_*j*_) maps in Fig. 8(b) show how the packing density increases over time, with the central region becoming progressively denser. The corresponding local orientation (*θ*^(*j*)^_6_) maps in Fig. 8(c) reveal the orientational order, showing multiple domains with different orientations at intermediate times, and eventually a single large domain with uniform orientation at later times. The continuous rotation allows for dynamic reorganisation and rapid grain-boundary migration, much faster than in non-driven systems.^16^ As the rotation continues, the small grains gradually merge and reorient, eventually forming a single crystalline domain within the LG beam, as shown in Fig. 8 (a, 117 min). Beyond studying crystallisation and grain growth in rotationally driven systems, this method could also be applied to binary colloidal fluids, where processes such as fractionation and glass formation become important.

## 5 Conclusions

We have introduced a method based on holographically generated defocussed Laguerre–Gaussian beams to create well-defined grain boundary loops with radii ranging from ∼5 to 100 particle diameters in two-dimensional colloidal crystals. We characterised the axial intensity distribution of the LG beam and demonstrated how applying LG beams at different distances from the focal point affects the colloidal crystal. We further characterised the effect of laser power and showed how LG beams with different vortex numbers (topological charges) can be used to create grain boundary loops with different radii. Finally, we compared our method with an earlier approach based on a combined focussed LG beam and defocussed Gaussian beam, and showed how the present defocussed LG beam method can be used to study crystallisation and grain growth in rotationally driven colloidal systems.

## Author contributions

Conceptualisation: HM, BvdM, MGB, AC, RPAD; formal analysis: HM, SC, AC; funding acquisition: BvdM, RPAD; investigation: HM, SC, AC; methodology: HM, SC, BvdM, MGB, AC; supervision: AC, RPAD; writing – original draft: HM, AC, RPAD; writing – review and editing: all authors.

## Conflicts of interest

There are no conflicts to declare.

## Data Availability

All the data supporting the findings of this study are included in the article and is available upon reasonable request.
